# Supplementary material for: Stem cell transplantation rescued a primary open-angle glaucoma mouse model
Source: eLife. 2021 Jan 28;10:e63677. doi: 10.7554/eLife.63677 (PMC7864631; doi:10.7554/eLife.63677)
Supplement: Supplementary file 1. — Related to Figure 9. [file elife-63677-supp1.docx]

**Supplementary File 1.** Related gene expression increase (p<0.05) in genes related to TM ECM interaction. Related to Figure 9.

|  | **Fibro-3** | **Fibro-2** | **Fibro-1** | **TMSC-3** | **TMSC-2** | **TMSC-1** |
| --- | --- | --- | --- | --- | --- | --- |
| **VTN** | 39.13535 | 26.4968 | 52.68634 | 36.58226 | 207.3045 | 654.0196 |
| **COL4A5** | 1822.884 | 852.9932 | 1141.186 | 7856.286 | 8172.403 | 3951.806 |
| **MYLK** | 2636.487 | 2302.165 | 1633.277 | 1357.498 | 397.1032 | 4418.69 |
| **PDGFD** | 2599.411 | 868.2799 | 2079.003 | 125.5661 | 3435.726 | 5045.021 |
| **COL4A6** | 189.4975 | 66.24201 | 80.08324 | 4491.708 | 5919.694 | 1349.094 |
| **HSPG2** | 11474.9 | 9403.308 | 17417.05 | 6801.333 | 8018.537 | 17596.47 |
